# Systematic Analysis of the Role of RNA-Binding Proteins in the Regulation of RNA Stability

**DOI:** 10.1371/journal.pgen.1004684

**Published:** 2014-11-06

**Authors:** Ayesha Hasan, Cristina Cotobal, Caia D. S. Duncan, Juan Mata

**Affiliations:** Department of Biochemistry, University of Cambridge, Cambridge, United Kingdom; The University of North Carolina at Chapel Hill, United States of America

## Abstract

mRNA half-lives are transcript-specific and vary over a range of more than 100-fold in eukaryotic cells. mRNA stabilities can be regulated by sequence-specific RNA-binding proteins (RBPs), which bind to regulatory sequence elements and modulate the interaction of the mRNA with the cellular RNA degradation machinery. However, it is unclear if this kind of regulation is sufficient to explain the large range of mRNA stabilities. To address this question, we examined the transcriptome of 74 *Schizosaccharomyces pombe* strains carrying deletions in non-essential genes encoding predicted RBPs (86% of all such genes). We identified 25 strains that displayed changes in the levels of between 4 and 104 mRNAs. The putative targets of these RBPs formed biologically coherent groups, defining regulons involved in cell separation, ribosome biogenesis, meiotic progression, stress responses and mitochondrial function. Moreover, mRNAs in these groups were enriched in specific sequence motifs in their coding sequences and untranslated regions, suggesting that they are coregulated at the posttranscriptional level. We performed genome-wide RNA stability measurements for several RBP mutants, and confirmed that the altered mRNA levels were caused by changes in their stabilities. Although RBPs regulate the decay rates of multiple regulons, only 16% of all *S. pombe* mRNAs were affected in any of the 74 deletion strains. This suggests that other players or mechanisms are required to generate the observed range of RNA half-lives of a eukaryotic transcriptome.

## Author Summary

Messenger RNAs (mRNAs) are the molecules that relay the information from genes (DNA) to proteins. Cells contain different amounts of each mRNA type depending on their function and their situation. The quantity of each mRNA depends on the balance between its production (transcription) and its degradation (mRNA decay). Recent studies have shown that the rate at which each mRNA is degraded is specific for every gene, but little is known about how this is regulated. In this work, we look at the role of a class of proteins that bind to RNA molecules (RNA-binding proteins, or RBPs) in the regulation of RNA decay. By systematically examining cells in which a single RBP has been inactivated we identify those that are important for RNA degradation. We found RBPs that make mRNAs more stable (that is, they are degraded more slowly) and others that make them unstable. These RBPs control the RNAs of genes with common features, suggesting that they provide a way of coordinating the function of groups of genes. However, for many genes we did not find RBPs that control their stability, indicating that other players are important to regulate RNA degradation.

## Introduction

The steady state levels of messenger RNAs (mRNAs) are determined by both their synthesis and decay rates [1]. Moreover, decay rates determine the time required for a new steady state to be reached after changes in transcription, and thus contribute to shaping dynamic changes of mRNA levels [2]–[5]. mRNA decay rates are transcript-specific [6], [7], and vary over a range of up to 100-fold [8]. In the budding yeast *Saccharomyces cerevisiae*, mRNA half-lives vary from a few minutes to over 3 hours (with medians between 12 and 23 minutes depending on the method used for their determination) [9]–[11]. The fission yeast *Schizosaccharomyces pombe* displays similar variations, with a median of 30 to 60 minutes [10], [12]. mRNAs in mammalian cells are typically longer-lived, with half-lives ranging from less than 20 minutes to several days [13]–[16].

Eukaryotic mRNAs are protected from exonuclease degradation by the 5′ methylated guanosyl cap and the 3′ poly(A) tail, which is coated with poly(A)-binding protein. In the most common pathway, degradation starts with the removal of one or both of these protective structures, followed by digestion through the action of 5′→3′ or 3′→5 exonucleases. The enzymatic activities associated with cytoplasmic mRNA decay are performed by a small number of protein complexes, most of which are conserved from yeast to humans. Deadenylation is carried out by three different deadenylases (Ccr4-Not, PAN2/PAN3 and PARN), decapping by the DCP1/DCP2 heterodimer, 5′→3′ degradation by the Xrn1 exonuclease, and 3′→5′ degradation by the cytoplasmic exosome [8], [17]. Xrn1-mediated 5′→3′ decay appears to be the dominant pathway in *S. cerevisiae* [18], [19].

The different steps of mRNA decay (deadenylation, decapping, and exonuclease degradation) take place with transcript-specific rates, and thus contribute to determine the overall decay rate [20], [21]. The stability of a specific transcript is at least partly controlled by *cis*-acting sequences, which are frequently – but not always – located in 3′ untranslated regions (UTRs) [22], [23]. These sequence motifs act by binding to sequence-specific RNA-binding proteins (RBPs), which in turn modulate the interaction of the mRNA with elements of the core degradation machinery (see [8] for a review). For example, many mammalian mRNAs contain sequences called AREs (AU-rich Elements) in their 3′ UTRs that make them unstable. Although the exosome is required for the rapid degradation of these mRNAs, it cannot recognize them on its own [24]. Instead, a number of ARE-binding proteins associate with these sequence elements and recruit components of the core degradation machinery, including decapping enzymes, the Ccr4-Not deadenylase complex, the exosome and Xrn1 [25]. Similarly, the budding yeast protein Puf5, a member of the pumilio family of RNA-binding proteins, recruits the Ccr4-Not complex and components of the decapping complex to mRNAs [26]. Other ARE-binding proteins, such as HuR, stabilize their targets. Although the exact molecular mechanisms are unclear, this effect could be mediated through protection from miRNA-mediated degradation [27]. However, many unicellular organisms lack the machinery for the production of small RNAs (such as *S. cerevisiae*) [28] or have it but do not use it for the control of mRNA stability (*S. pombe*) [29].

It is becoming apparent that mRNA degradation and transcription are intimately linked. This was suggested by early work that showed that mutants in the *S. cerevisiae dcp1* gene (encoding a component of the decapping complex) affected RNA stability without causing changes in RNA levels, possibly indicating compensatory changes in transcription [30]. More recently, several studies of RNA synthesis and decay rates have revealed widespread decreases in transcription rates in response to the inactivation of multiple RNA decay pathways such as the Ccr4-Not complex, Xrn1, and the exosome [10], [31], [32]. Xrn1 appears to have a key function in this feedback by directly stimulating transcription [31], [32].

The fission yeast *Schizosaccharomyces pombe* provides an attractive model for the study of posttranscriptional regulation in eukaryotic organisms. In addition to the major pathways described above, *S. pombe* contains decay-related components that are present in higher eukaryotes but not in *S. cerevisiae*, such as cytoplasmic poly(A) polymerases [33], a poly(A)-specific ribonuclease (PARN) [34] and a poly(U) polymerase-dependent decay pathway [34].

It is still unclear how the specificity of decay rates is determined. Although RBP-mediated recruitment of the decay machinery can modulate the decay rates of individual mRNAs, it is not known if the large range in mRNA half-lives can be explained exclusively by this mechanism. To investigate this issue we performed gene expression profiling for 86% of *S. pombe* deletions in non-essential genes encoding RBPs. We found 25 strains that showed significant changes in RNA levels, affecting between 4 and 104 mRNAs. In addition, we identified 4 strains with defects in splicing. The potential targets of these RBPs had common properties, such as being coexpressed in response to stress, or encoding proteins with similar localization or involved in the same pathway. Unexpectedly, only 16% of all *S. pombe* mRNAs showed changes in levels in at least one of the 74 strains tested. This suggests that the action of these RBPs is not sufficient to explain the large range of mRNA half-lives in fission yeast, and that additional mechanisms may be required.

## Results

### Selection of RBPs

We focused on proteins that contained RNA-binding domains that might confer sequence-specificity, although we also investigated a few proteins with catalytic activities on RNA (such as nucleases). Table 1 lists the 16 domains we selected with their PFAM references, and Table S1 presents all the selected proteins. The most common domains were the RRM (RNA recognition motif, present in 72 proteins in fission yeast), PUF (Pumilio Family RNA binding repeat, in 9 proteins), several zinc finger domains (a total of 20 proteins), the KH (K-Homology domain, 8 proteins) and the G-patch domain (8 proteins). Seven proteins had domains from more than one family, and a total of 136 predicted proteins contained at least one of the selected domains. Of the genes encoding these proteins, 47 were essential, 86 were non-essential, and for the remaining 3 there was no information or conflicting data [35], [36]. Therefore, the fraction of essential genes encoding RBPs in *S. pombe* is 34.5%, which is significantly higher than the overall percentage of essential genes (26% for all genes, p-value 6.5×10^−3^). For the majority of the strains we used the Bioneer gene deletion library [35] (see Table S11 for full details). The correct deletion of the genes was assessed from the microarray data and/or by gene-specific PCR. We confirmed the deletion was present in 74 genes out of our 86 target genes (86%), while in 12 strains the gene was not deleted (Table S1).

**Table 1:** RNA-binding domains considered in this study.

Domain	Full name	PFAM Reference	Total	Essential	Non-essential	Unknown or ambiguous	Analysed	Not deleted
**RRM**	RNA Recognition Motif	PF14259	72	25	45	2	37	8
		PF00076						
**PUF**	PUmilio-Family RNA binding repeat	PF00806	9	1	8	0	8	0
**zf-CCCH**	zinc finger C-x8-C-x5-C-x3-H type	PF00642	9	5	4	0	4	0
**KH**	K-Homology domain	PF00013	8	3	4	1	2	2
		PF13083						
		PF13014						
**zf-CCHC**	zinc Knuckle	PF00098	8	4	4	0	3	1
		PF13696						
		PF14392						
**G-patch**	G-patch domain	PF01585	8	1	7	0	7	0
**Brix**	Brix domain	PF04427	5	5	0	0	0	0
**zf-U1**	U1 zinc finger	PF06220	2	1	1	0	1	0
**FDF**	FDF domain	PF09532	2	1	1	0	1	0
**La**	La domain	PF05383	2	0	2	0	2	0
**zf-C3H1**	Putative zinc finger domain	PF10650	1	0	1	0	1	0
**TAP-C**	TAP C-terminal domain	PF03943	1	0	1	0	1	0
**RNA12**	RNA12 protein	PF10443	1	0	1	0	1	0
**Tudor**	Tudor	PF00567	1	0	1	0	1	0
**Endonuclease _NS**	DNA/RNA non-specific endonuclease	PF01223	1	0	1	0	1	0
**Unclassified**	Unclassified	NA	8	0	8	0	7	1
**S1**	S1 domain	PF00575	5	4	1	0	1	0
**Total (sums)**			143	50	90	3	78	12
**Total (unique proteins)**	*Note 7 proteins have more than 1 domain*		136	47	86	3	74	12

### Transcriptome analysis of deletion strains

**Figure 1:**
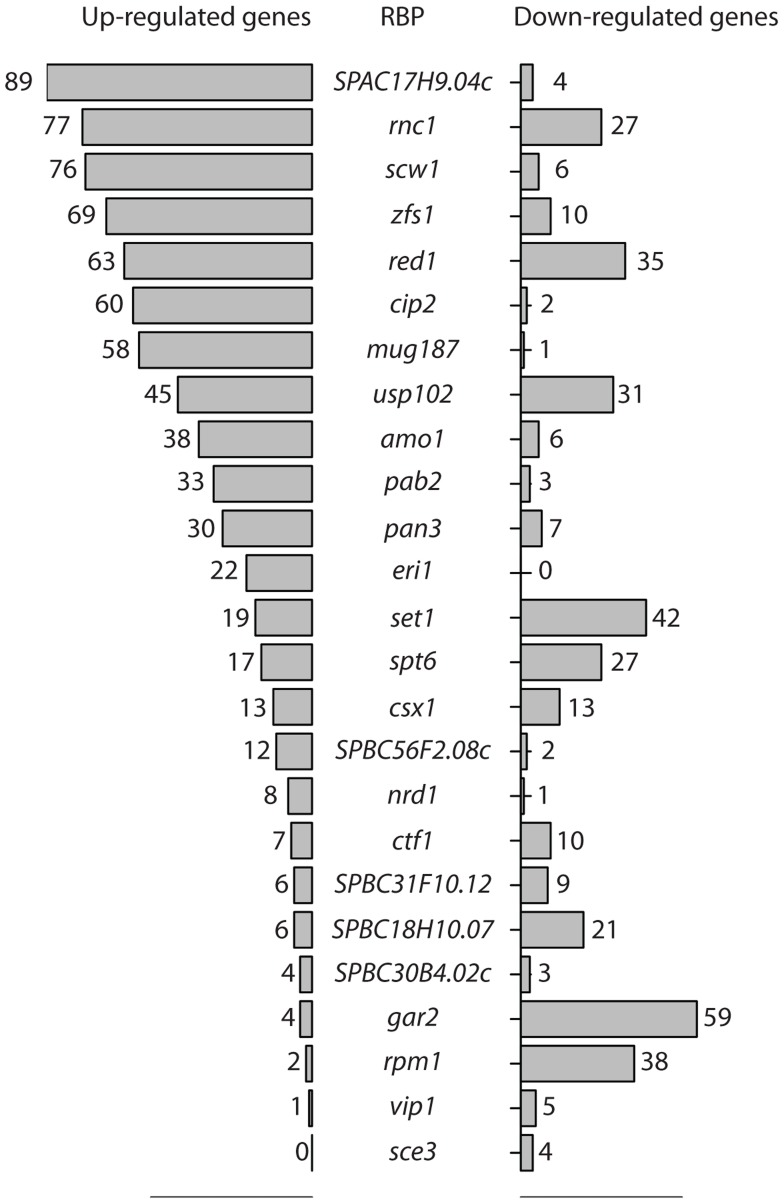
RNA-binding proteins whose inactivation causes changes in RNA levels. Numbers of RNAs whose expression is increased (bars on the left side) or decreased (bars on the right side) when the genes encoding the indicated RNA-binding proteins are deleted. Statistical significance was determined as described in Materials and Methods.

Changes in stability are expected to affect mRNA steady state levels. Therefore, to identify RBPs with a potential role in decay, we used custom-designed oligonucleotide microarrays to compare the transcriptome of each of the 74 deletion strains to that of isogenic wild type cells grown under vegetative conditions. The microarrays contained two probes for every annotated *S. pombe* coding sequence as well as for 496 long non-coding RNAs and 1,491 introns. We initially carried out two independent biological replicates for each strain, and performed a third repeat for those that showed changes in the first two experiments. We used a robust statistical method to identify differentially-expressed genes (Significance Analysis of Microarrays, or SAM) [37]. A total of 25 deletions caused significant changes in gene expression, with the numbers of affected genes ranging from 4 to 104 (Figure 1). The majority of strains showed mostly up-regulated genes or both up- and down-regulation, while only few strains displayed predominantly down-regulation (Figure 1). Complete lists of affected genes and their corresponding enrichment analysis are presented in Tables S2, S3, and S4.

To validate our approach, we initially focused on those genes for which genome-wide transcriptome data was available. The *zfs1* gene codes for a zinc-finger protein of the tristetraprolin family [38] and has been extensively studied at the genome-wide level. We found that *zfs1* deletion caused up-regulation of 69 genes, which showed a highly significant overlap with the 60 genes identified in a published study [39] (Figure S1A).

In fission yeast vegetative cells a subset of meiotic mRNAs are bound to a protein called Mmi1, which targets them for degradation by the nuclear exosome in a process that requires Pab2 (a nuclear poly(A)-binding protein) and Red1 (a Zinc finger-containing protein) [40]–[42]. *pab2* mutants showed up-regulation of 33 coding genes, while *red1* mutants overexpressed 63 mRNAs. The majority of mRNAs affected by the *pab2* deletion were also overexpressed in *red1Δ* cells, and the effect was stronger in the latter (average induction of 5.13, compared to 2.8 in *pab2Δ*). This suggests that both proteins regulate the same group of mRNAs, although the function of Red1 appears to be more important. Both sets of mRNAs showed strong overlap with mRNAs physically associated with Mmi1 (Caia Duncan and Juan Mata, unpublished data) and with published microarray data on *pab2* and *red1* mutants [41], [42] (Figure S1B and S1C).

**Figure 2:**
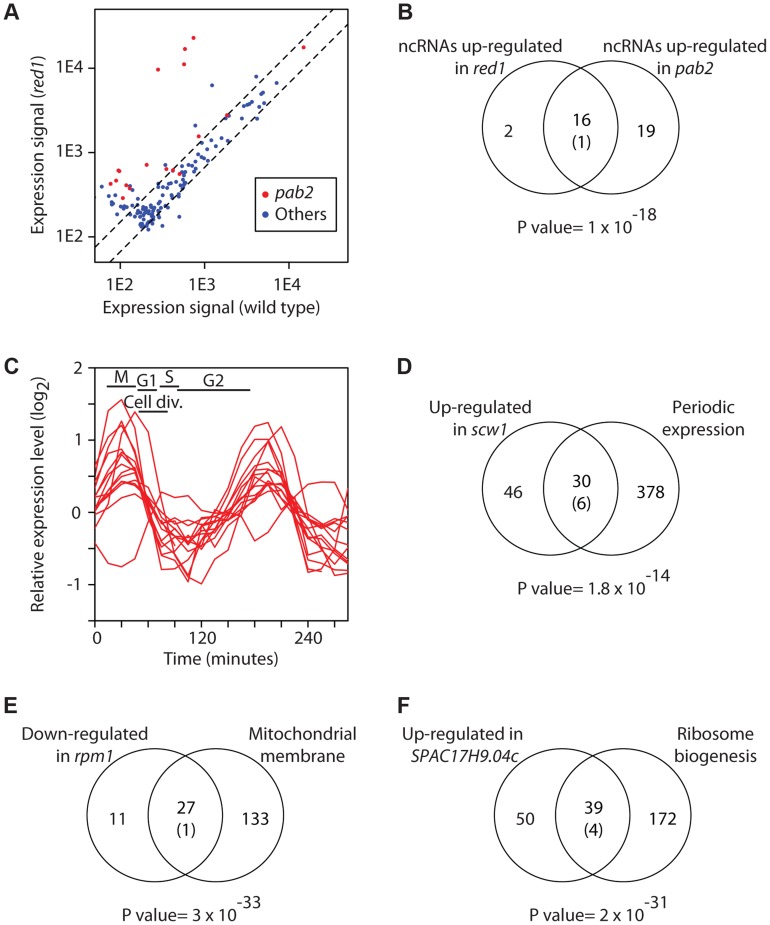
Characterization of the RNA sets regulated by RBPs. (A) Inactivation of *pab2* and *red1* causes overexpression of a subset of ncRNAs. The y and x axes show expression levels in *red1* mutants and wild type cells, respectively. Each dot corresponds to a different ncRNA. ncRNAs that are overexpressed in *pab2Δ* cells are displayed in red and all other ncRNAs in blue. (B) Similar sets of ncRNAs are affected in *pab2* and *red1* mutants. The Venn diagram shows the overlap among the ncRNAs overexpressed in *red1Δ* and *pab2Δ* cells. The number in brackets corresponds to the expected overlap if randomly-generated lists of the corresponding sizes were used. The p value of the observed overlap is shown below the Venn diagram. (C) Scw1 potential targets are enriched in cell cycle-regulated genes. Cell cycle expression profiles of periodically expressed Scw1-regulated genes (data from [76]). The timing of the cell cycle phases is shown: M (mitosis), G1, S phase (S), and G2. Cell division takes place during G1 and S phase. (D) Overlap between cell-cycle regulated genes and Scw1-regulated mRNAs. Labelling as in B. (E) Comparison of genes down-regulated in *rpm1* mutants and genes encoding proteins localized to the mitochondrial membrane. Labelling as in B. (F) Overlap between genes overexpressed in *SPAC17H9.04c* mutants and genes encoding proteins involved in ribosome biogenesis. Labelling as in B.

Pab2 is also involved in the processing of snoRNAs [43]. The presence of long ncRNA probes in our microarray platform allowed us to investigate if Pab2 and Red1 are involved in the regulation of other non-coding transcripts. Indeed, we found 18 ncRNAs overexpressed in *pab2Δ*, and 35 in *red1Δ* (Figure 2A and Table S5). As was the case with coding genes, almost all ncRNAs up-regulated in *pab2Δ* were also induced in *red1Δ* (Figure 2B and Table S5). Some of these ncRNAs were overexpressed more strongly than most coding targets, suggesting that they may be functionally important. These results demonstrate that our strategy can identify both known targets and additional functions of RBPs. While this manuscript was in preparation, a transcriptome analysis of *red1Δ* mutants using high throughput sequencing was published [44]. This study reported overexpression of large numbers of ncRNAs in *red1Δ* cells, which showed a highly significant overlap with our data (Figure S1D).

Below we discuss a few notable examples of RBPs identified in the screen. *scw1* encodes an RRM-containing protein and is essential for correct cell wall structure and cell separation. Mutants in *scw1* display multiple septa, indicating a defect in cell separation, but the molecular basis for this phenotype is unknown [45], [46]. *scw1* deletion caused the overexpression of 76 genes, which were enriched in periodically-expressed genes (Figure 2C and 2D) as well as in genes encoding proteins required for cell wall organization and cytokinesis. For example, the induced genes included *gas1*, *gas2* and *gas5*, encoding three 1,3-β-glucanosyltransferases [36], *bgl2*, which codes for a glucan exo-1,3-β-glucosidase [36], *pmk1*, encoding a MAP kinase that regulates cell wall integrity [47], and *rho2*, which codes for a GTPase that regulates cell wall alpha-glucan biosynthesis [48]. Our results suggest that overexpression and/or mis-expression of enzymes involved in cell wall biosynthesis are responsible for the cell separation phenotype of *scw1* mutants. Mutations in *scw1* supress defects in the SIN pathway, which regulates cytokinesis in *S. pombe* [45], [46]. Moreover, Scw1 is a target of the Sid2 kinase, a key effector of the SIN pathway, suggesting that the SIN pathway may regulate Scw1 function [49]. The putative targets of Scw1 included *mob2*, which encodes the regulatory subunit of Sid2, suggesting that the interactions between the SIN pathway and Scw1 may be complex. In addition, *scw1* mutants are released from a *cdc25* G2 cell cycle block with very poor synchrony, possibly indicating mitotic defects (JM and AH, unpublished data). It has been reported that *scw1* mutants fail to arrest in mitosis in *nda3* cold-sensitive mutants (encoding β-tubulin), probably because mutations in *scw1* lead to microtubule stabilization [45]. All together, these results suggest that Scw1 may have additional roles in cell cycle control.

*rpm1* (also called *par1*) encodes a protein with a predicted exonuclease II domain that localizes to mitochondria [50], [51]. Rpm1 is essential for growth in non-fermentable carbon sources, and *rpm1Δ* cells are defective in the processing of mitochondrially-encoded transcripts that encode key components of the respiratory chain, resulting in the accumulation of their RNA precursors [50]. *rpm1* deletion caused reduced expression of a set of 38 nuclear-encoded genes, most of which encoded proteins localized to the mitochondrial envelope, including multiple components of the F0 and F1 ATPases and the cytochrome c oxidase complex (Figure 2E). These genes showed a significant overlap with those under-expressed in cells lacking the m-AAA protease, which is involved in the processing of mitochondrial proteins [52]. Although the effect of Rpm1 on these mRNAs is likely to be indirect (given that, as an exonuclease, its deletion would be expected to lead to overexpression of its targets), it appears to be posttranscriptional (see below). The fact that two different mutations that affect the production of key mitochondrial protein complexes cause down-regulation of nuclear-encoded RNAs suggests the existence of a checkpoint mechanism that monitors the formation of respiratory complexes and responds by destabilising mRNAs encoding components of these complexes.

*SPAC17H9.04c* codes for a protein with two zinc finger domains and an RRM, which is localized to the nucleolus and cytoplasmic dots [51]. *SPAC17H9.04c*Δ cells overexpressed 89 genes, ∼44% of which encoded proteins localized to the nucleolus and/or involved in ribosome biogenesis (Figure 2F). These included 10 U3 snoRNA-associated proteins, 4 nucleolar ATP-dependent helicases, and several rRNA-modifying enzymes. In addition, mRNAs encoding proteins involved in rRNA processing, but not localized to the nucleolus, were also induced, suggesting that these genes define a novel regulon involved in rRNA maturation.

*mug187* encodes a predicted protein containing two RRMs that is induced during meiosis [53] and in response to multiple stress conditions, including cadmium treatment, heat shock and osmotic shock [54]. A total of 66 genes were induced in *mug187* mutants (grown in the absence of stress), which were enriched in genes specifically induced in response to cadmium, and in genes encoding enzymes required for serine and sulphur aminoacid biosynthesis.

Several mutants showed up-regulation of ncRNAs (Table S5). Of particular interest was *pan3Δ*, which encodes a subunit of the Pan2/Pan3 complex. This complex has poly(A)-specific ribonuclease activity and is thought to regulate poly(A) tail length. Our results indicate that Pan2/Pan3 is required for the down-regulation of stress genes (Table S3) and ncRNAs (Table S5). This is, to our knowledge, the first indication that this complex is involved in the degradation of ncRNAs.

As discussed above, mRNA half-lives are transcript-specific and vary over a range of more than a hundred fold. To evaluate the contribution of non-essential RBPs to this phenomenon we quantified the fraction of genes whose expression was affected in at least one RBP mutant. A total of 816 genes were differentially expressed in at least one of the 74 strains we characterised (529 only up-regulated, 287 only down-regulated, and 46 induced in some strains and repressed in others). These genes represent only 16.2% of all coding sequences. This result suggests that even though non-essential RBPs influence the stability of several coherent sets of genes, their function is not sufficient to explain the large range in decay rates of the fission yeast transcriptome.

### Effects on splicing and pre-mRNA decay

Our array platform contains probes for 1,491 introns (out of 4,730 in the *S. pombe* genome), allowing the identification of strains with deficient splicing. Splicing defects are expected to lead to the accumulation of pre-mRNAs and thus to overexpression of intronic regions. Only a few hundred introns were detected in most experiments, consistent with their short half-lives and general low levels (see below).

**Figure 3:**
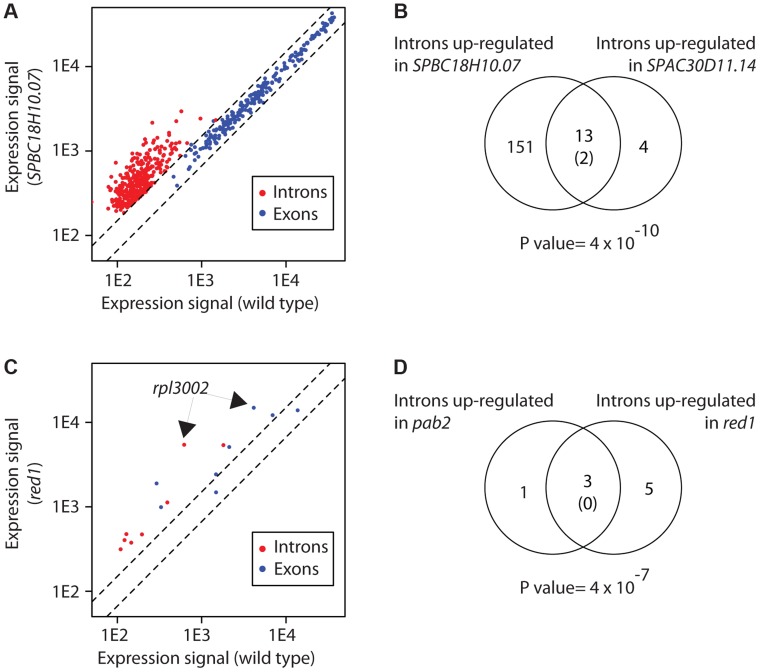
Changes in intron levels caused by RBP inactivation. (A) Transcriptome of *SPBC18H1.07* mutant cells. The y and x axes show expression levels in mutant and wild type cells, respectively. Each dot corresponds to a different gene. Introns overexpressed in the mutant are shown in red, and the coding sequences of the corresponding genes in blue. Note that introns of the affected genes, but not their exons, are affected. (B) Overlap between introns overexpressed in *SPBC18H10.07* and *SPAC30D11.14* mutants. The number in brackets corresponds to the expected overlap if randomly-generated lists of the corresponding sizes were used. The p value of the observed overlap is shown below the Venn diagram. (C) Transcriptome of *red1* mutants. Labelling as in A. Introns overexpressed in *red1* cells are shown in red, and the corresponding coding sequences in blue. Note that both introns and exons of the affected genes are frequently overexpressed. The arrows mark the position of the *rpl3002* exon and intron probes. (D) Overlap between introns overexpressed in *pab2* and *red1* mutants. Labelling as in B.

We observed splicing defects in strains that carry mutations in genes encoding predicted components of the core splicing machinery. *usp102* encodes a U1 snRNP-associated protein that contains an RRM, and *SPBC18H10.07*, a U1-type zinc finger predicted to bind to the U1 snRNA [36]. *usp102* deletion had a modest effect with 25 overexpressed introns, while *SPBC18H10.07* inactivation led to the accumulation of 268 introns (Table S6). In both cases, exonic probes of the corresponding genes did not detect any overexpression, suggesting that the increase in introns was due to the accumulation of unspliced pre-mRNAs, and not to a general increase in transcript abundance (Figure 3A). Both sets of introns overlapped extensively, suggesting that both proteins regulate the splicing of the same mRNA set. We also identified novel proteins with a function in splicing. *SPAC30D11.14c*, which encodes an uncharacterised RBP that contains a KH domain [36], overexpressed 50 introns (Figure 3B and Table S6). We performed GO-enrichment analysis of the corresponding genes, but could not find any specific enrichment. However, those introns overlapped significantly with those identified in *usp102* and *SPBC18H10.07* mutants (Figure 3B), suggesting that they may represent a set of introns whose splicing is sensitive to decreased efficiency of the splicing machinery.

The increase in RNA levels detected by intronic probes could also be the result of a failure to degrade spliced introns (rather than an accumulation of unspliced pre-mRNAs). To address this possibility we performed RNA-seq experiments with mutants in *SPBC18H10.07* and *SPAC30D11.14c*. We expected that a splicing defect would lead to an increase in both reads mapping to introns and to exon-intron junctions, whereas a defect in intron turnover would cause an accumulation in intronic reads but not in exon-intron junctions (Figure S2A). Both mutants showed a build-up of intronic reads in specific sets of introns that overlapped significantly with the introns identified using microarrays (p values of 6×10^−47^ and 8×10^−7^ for *SPBC18H10.07* and *SPAC30D11.14c*, respectively). Moreover, this increase was accompanied by an accumulation of reads mapping to the corresponding exon-intron junctions, indicating that the mutations cause inefficient splicing of pre-mRNAs (Figure S2B–E).

Another gene, *SPAC23A1.09*, accumulated 15 introns (but not the corresponding exons), which did not overlap with those increased in other mutants (Table S6). *SPAC23A1.09* encodes the fission yeast ortholog of Y14 (also known as RNA-binding protein 8), which is a component of the exon junction complex (EJC), a multiprotein complex that is deposited upstream of exon-exon junctions and that has functions in RNA export, translational control and nonsense-mediated decay (NMD) [55]. Although the components of the EJC have not been studied in fission yeast, the EJC does not appear to have a function in NMD [56]. Our results suggest that the EJC may have a role in enhancing splicing efficiency for a subset of pre-mRNAs.

Mutations in *pab2Δ* lead to the increased expression of both intronic and exonic regions of 21 genes [57]. In-depth biochemical analysis of this phenomenon using the *rpl3002* transcript as a model revealed that Pab2 and the nuclear exosome are part of a polyadenylation-dependent pre-mRNA degradation pathway. We identified 4 genes with increased intronic expression in *pab2Δ* (Table S6), and 2 of these were also up-regulated at the exonic level. The only gene in common with the previous study was *rpl3002*. The reason for this discrepancy is unclear. It is possible that our data processing was more stringent, as our lists have been derived from a larger number of biological repeats. On the other hand, microarrays do not have complete coverage of all introns and are also less sensitive. In the case of Red1, which cooperates with Pab2 in nuclear RNA degradation, we found 8 introns overexpressed in *red1Δ* cells (Figure 3C). Interestingly, the group of 8 introns contained 3 of the 4 introns up-regulated in *pab2Δ* cells (Figure 3D). By contrast with *usp102* and *SPBC18H10.07*, the overexpression of 6 introns in *red1Δ* cells was accompanied by increased levels of the corresponding exonic probes (Figure 3C). These observations suggest that Red1 has a role in the Pab2-dependent pre-mRNA decay pathway. Mutants in other potential regulators of RNA decay identified in this screen did not show concurrent overexpression of introns and exons, indicating that they regulate the degradation of mature mRNAs.

Finally, *pab2Δ* and *red1Δ* mutants showed reduced expression of 21 and 18 introns, respectively, that showed a significant overlap and was not accompanied by a decrease in the corresponding exonic sequences. Therefore, Pab2 and Red1 appear to be required for the efficient splicing of a group of genes.

### Transcriptional vs posttranscriptional effects

The RBPs analysed in this work may affect transcription, either directly or indirectly through changes in RNA levels of transcription factor genes. Therefore, an important question is whether the observed changes in expression are due to alterations in transcription or RNA decay rates. To address this issue we measured RNA decay rates at the genome-wide level for nine selected strains that showed clear changes in RNA levels. We selected a protein known to regulate chromatin structure (Set1, which contains a single RRM) and eight additional proteins (Red1, Pab2, Zfs1, SPAC17H9.04c, Rpm1, Csx1, Scw1, and Rnc1).

To measure RNA stabilities we used an approach based on the *in vivo* labelling of RNAs with the modified nucleoside 4-thiouridine (4sU) [8], [12]. Cells are incubated with 4sU, which is incorporated into newly synthesised RNA at a rate determined by the stability of the RNA. As long as the system is under steady-state conditions (that is, if RNA levels do not change over time), the fraction of labelled mRNA for a given gene can be used to estimate its decay rate. To measure this fraction, total RNA is prepared and 4sU–labelled RNA is specifically biotinylated. The biotinylated RNA can then be purified using streptavidin magnetic beads, and is compared to total RNA using two-colour custom DNA microarrays.

**Figure 4:**
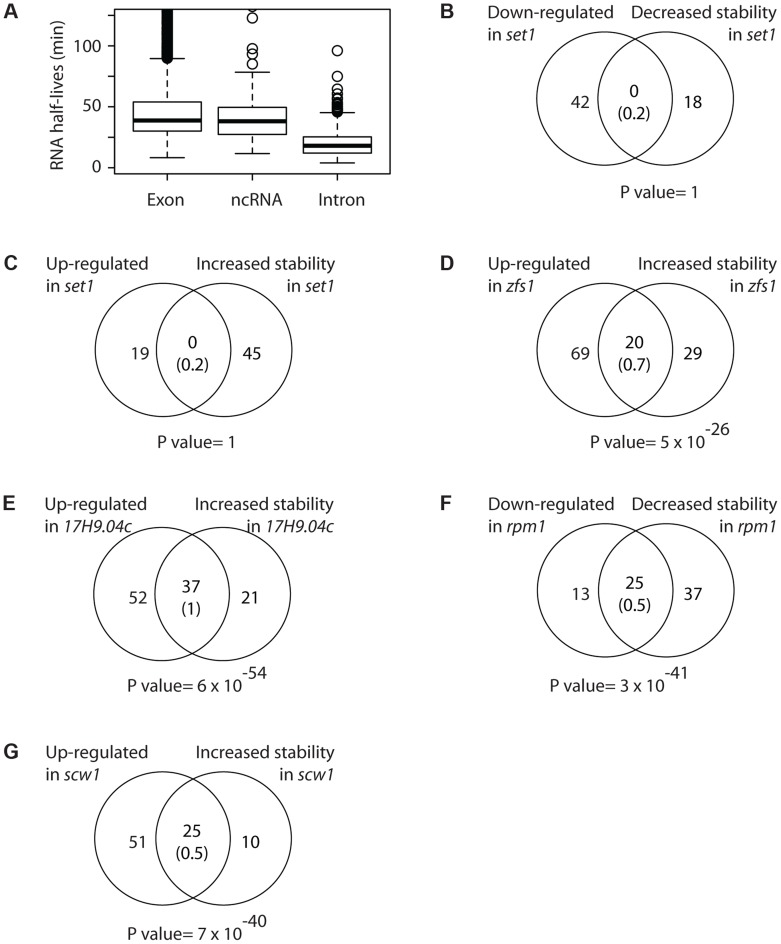
Correlations between changes in mRNA stability and mRNA levels. (A) Boxplot showing the distributions of half-lives for mRNAs (left), ncRNAs (middle) and introns (right). (B) Overlap between mRNAs down-regulated and destabilized in *set1* mutants. The number in brackets corresponds to the expected overlap if randomly-generated lists of the corresponding sizes were used. The p value of the observed overlap is shown on the right side. (C) As in B, comparing mRNAs up-regulated and stabilized in *set1Δ* cells. (D) As in B, for mRNAs up-regulated and stabilized in *zfs1* mutants. (E) As in B, for mRNAs overexpressed and stabilized in *SPAC17H9.04c*. (F) As in B, for mRNAs down-regulated and destabilized in *rpm1* mutants. (G) As in B, for mRNAs up-regulated and stabilized in *scw1* mutants.

We have previously set up this approach for *S. pombe* and showed that the steady state assumption holds for the conditions we employ [12]. As part of these experiments we measured RNA stabilities for 7 independent wild type samples. Reproducibility was high among biological replicates, with an average coefficient of variation of 12.6%. This high quality dataset improves and refines our previous estimates of half-lives (Table S7). Moreover, we provide the first quantification of stabilities of a subset of ncRNAs and introns in *S. pombe* (Table S7). Both ncRNAs and coding sequences showed similar half-lives (medians of 30.5 and 31 minutes, respectively), while introns were shorter-lived (14.5 minutes) (Figure 4A).

As expected, genes affected in *set1Δ* mutants did not show changes in stability (Figure 4B and 4C). By contrast, Zfs1 targets predicted from the expression analysis were highly enriched among those mRNAs that displayed increased half-lives in the *zfs1Δ* mutant (Figure 4D).

Red1 and Pab1 are part of a system that down-regulates the expression of a group of meiotic mRNAs in vegetative cells, which are strongly overexpressed in *red1Δ* and *pab2Δ* mutants. We observed highly significant overlaps between mRNAs with increased stabilities in the mutants and those overexpressed, confirming and extending the observations that these proteins promote the decay of their mRNA targets (Figure S3A and S3B). Moreover, ncRNAs induced in *red1* mutants (see above) also displayed extended half-lives, indicating that Red1 promotes the decay of multiple ncRNAs.

The effect of *SPAC17H9.04c*, which controls a regulon of genes involved in ribosomal synthesis, was also clearly posttranscriptional, as genes overexpressed in the mutant also showed enhanced stability (Figure 4E). Similarly, the under-expression of genes encoding mitochondrial components in *rpm1* mutants was strongly correlated with decreased stability (Figure 4F). Finally, *scw1* mutants showed correlated changes in mRNA stability and mRNA levels (Figure 4G).

By contrast, we did not observe stability changes for two RBP mutants, *rnc1* and *csx1* (Figure S3C and S3D). Csx1 regulates RNA stability in response to oxidative stress [58], while Rnc1 stabilises at least one mRNA in response to osmotic stress [59]. The reasons for this lack of correlation are unclear. It is possible that our system is not sensitive enough to detect very small changes in decay rates, and the changes in RNA levels in both of these mutants were subtle. Consistent with the complexity of the experiment to measure decay rates, it is our experience that this method is less sensitive than direct measurement of RNA levels. Alternatively, it is possible that Csx1 and Rnc1 only regulate RNA stability under conditions of stress.

Recent studies have shown that changes in RNA stability may be compensated by alterations in transcription rates [31]. To investigate if this phenomenon is prevalent in our system we measured RNA stability for three RBP mutants that did not display changes in mRNA levels: *SPAC25G10.01*, *SPAC16E8.06c* (*nop12*), and *SPAC683.02c*. In all three cases the fraction of mRNAs with altered RNA stability was between 0 and 0.04%, indicating that compensatory changes do not play an important role in these three strains.

### Identification of potential regulatory motifs

**Figure 5:**
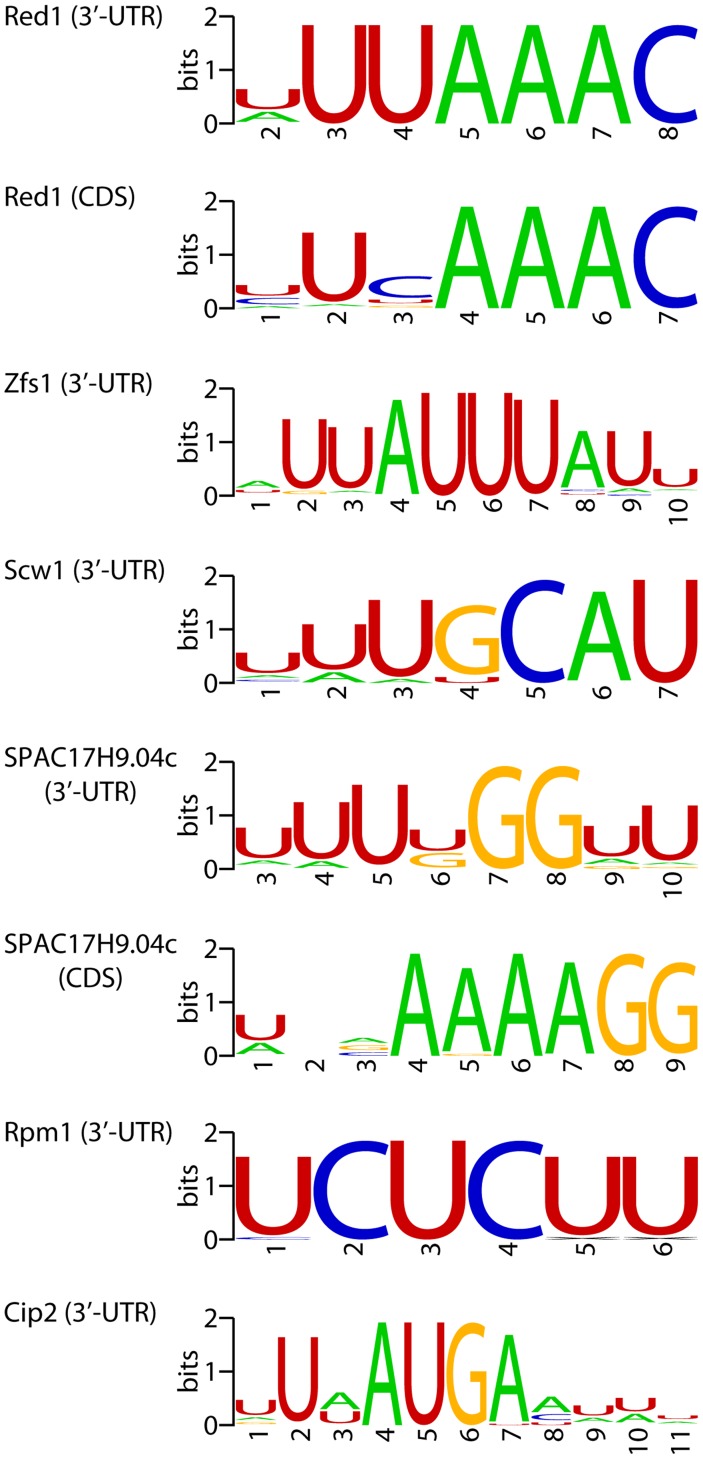
Potential regulatory elements. Sequence motifs enriched in mRNAs affected by deletions in the specified genes. Motifs were identified in coding sequences or 3′-UTRs as indicated. The sequence logos show the conservation at each position measured in bits (overall height of the stack), and the height of the symbols within the stack is proportional to the relative abundance of the bases.

**Figure 6:**
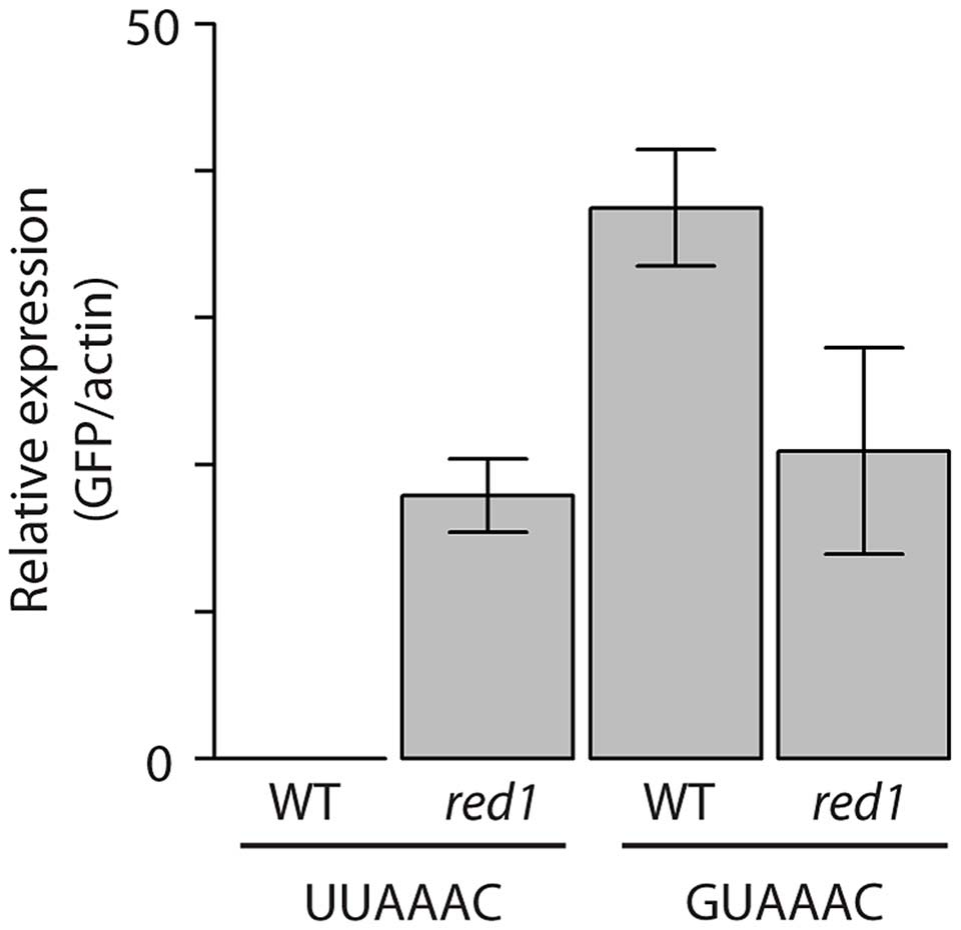
Functional validation of the *red1*-associated motif. Wild type or *red1Δ* cells were transformed with GFP reporter plasmids containing 8 copies of the *red1*-linked motif (UUAAAC) or a mutated version (GUAAAC). GFP RNA levels were estimated by qPCR and normalized to those of actin. The bars show the mean value of three independent biological repeats, and the error bars correspond to the standard deviation.

If a set of mRNAs is coregulated by a given RBP, it would be expected that the binding site of the RBP would be enriched in their sequences. Therefore, we searched for over-represented sequence elements in 5′-UTRs, 3′-UTRs and coding regions using software specifically designed for this purpose (REFINE, [60]). We identified potential regulatory sequences in 12 regions corresponding to 8 proteins (Table S8 and Figure 5). Eight of the predicted binding sites were located in the 3′-UTRs, while 3 were identified in coding regions and one in 5′-UTRs. Red1 potential targets were enriched in sequences related to the Mmi1-binding site [61] in both their coding regions and 3′-UTRs. Similarly, we found that genes up-regulated in *zfs1* mutants contained potential regulatory elements related to the published Zfs1 recognition site in both their 5′- and 3′-UTRs [39]. By contrast, mRNAs affected in *SPAC17H9.04c*Δ mutants were enriched in different sequence elements in their 3′-UTRs and coding regions. Surprisingly, the latter showed a very strong positional effect, with ∼67% of the sites located at positions 16 or 43 within the coding sequence. Finally, other RBPs that regulate groups of mRNAs with shared properties (Rpm1, Cip2 and Scw1) also contained enriched sequence elements (Figure 5). We chose the *red1*-enriched motif for further functional validation. A reporter construct [61] containing 8 tandem copies of the *red1* potential regulatory motif (UUAAAC) in its 3′ UTR was not detectable by qPCR, while one with a mutated motif (GUAAAC) was clearly expressed (Figure 6). Deletion of *red1* caused the UUAAAC reporter to be expressed at levels very similar to those of the reporter containing the mutated sequence (Figure 6), demonstrating that Red1 regulates mRNAs that contain the motif identified in our bioinformatics analyses.

### Direct vs indirect effects

Inactivation of an RBP can cause changes in the stability of its targets, but may also affect other genes in an indirect manner. To investigate this question we used RIp-chip (Ribonucleoprotein Immunoprecipitation analysed with DNA chips) to identify RNAs bound to three RBPs identified in this screen: Red1, Zfs1, and Scw1. The three proteins were epitope-tagged, immunopurified, and the associated RNAs identified using DNA microarrays (Table S9).

In all three cases the RNAs identified by RIp-chip displayed highly significant overlaps with those RNAs overexpressed in the corresponding RBP mutants (Figure S4). By contrast, there was no enrichment in mRNAs under-expressed in the mutants, strongly suggesting that the three proteins destabilize the RNAs they bind to, and that decreases in gene expression associated with the mutations are indirect (Figure S4). Surprisingly, a substantial number (∼50) of RNAs bound by Zfs1 and Scw1 overlapped (Table S9). However, these RNAs were not enriched in those associated with Red1 or other any *S. pombe* proteins that we had previously investigated by RIp-chip [12], [62]–[66]. Given the specificity of this overlap, it seems unlikely to represent a technical artefact, and could be explained if the two proteins are located to the same subcellular structure that co-purifies with them.

### Phenotypic characterisation of RBP mutants

It is possible that some RBPs do not have a role in vegetative cells and only function in specific developmental or environmental situations. For example, we have previously shown that the RBP Meu5 regulates mRNA stability during meiosis [12]. In vegetative cells, however, Meu5 is not expressed, and deletion of *meu5* did not cause any changes in the transcriptome. To investigate if some of these RBPs have functions in specialised situations we monitored the ability of the RBP mutants to grow in 11 different conditions: low and high temperature, using non-fermentable carbon sources (galactose), in the presence of hydroxyurea (an inhibitor of DNA synthesis), calcofluor (which impairs cell wall formation), cycloheximide (an inhibitor of translation), methyl benzimidazol-2-yl-carbamate (MBC, a microtubule poison), caffeine (which overrides the S/M checkpoint), H_2_O_2_ (that causes oxidative stress), cadmium (a heavy metal), methyl methanesulfonate (MMS, which induces DNA damage) and high concentrations of KCl (to trigger osmotic stress). All phenotypes were assessed by drop assays (Figure S5 and Table S10). In addition, we investigated the ability of the strains to undergo sexual differentiation (mating and spore formation, Figure S6 and Table S10). As a control we used a strain carrying a deletion in the *caf1* gene, which encodes one of the catalytic subunits of the Ccr4-Not complex, and which has been reported to be sensitive to several stresses [67], [68]. As expected, *caf1Δ* cells exhibited phenotypes in 9 different conditions (Table S10). 39 strains (53% of the collection) showed at least one phenotype (Figure S5). However, only nine displayed three phenotypes or more, and the four strains that showed most phenotypes were affected in genes involved in general expression pathways such as mRNA deadenylation (*caf1*), splicing (*usp102*), translation initiation (*sce3*) and nucleosome remodelling (*spt6*). These data indicate that the screen is highly specific. The most common phenotype was defects in sexual differentiation, which affected 16 strains (22%). Sensitivity to cadmium, caffeine and cycloheximide were also common (∼15%), the latter suggesting that some of these RBPs may have functions in translation. Interestingly, we found three strains that were strongly resistant to cadmium. The sensitivity to other environmental stresses was highly specific. For example, only three strains were sensitive to oxidative stress (*caf1*, *rpm1* and *csx1*), and a single one was unable to grow using a non-fermentable carbon source (*rpm1*). Altogether, our results demonstrate that many of the RBPs studied here have functions in the response to specific stress conditions and during cellular development.

## Discussion

We have systematically characterised the vegetative transcriptome and the function of the majority of genes encoding sequence-specific RBPs in *S. pombe*. Our results offer new biological insights and provide a valuable resource for the study of posttranscriptional control in fission yeast.

The characterisation of some previously studied genes provides new clues about their function. For example, we show that Red1 participates in a pre-mRNA degradation pathway, probably in cooperation with Pab2. Moreover, we have found that Red1 regulates the expression of long ncRNAs through the control of their stability. We have also identified new pathways (Pan2/Pan3) involved in the regulation of ncRNA expression.

Our systematic study has identified and characterised RBPs that regulate mRNA decay in *S. pombe* cells and has provided lists of their potential targets. The RBP putative targets presented common features, such as being co-expressed in response to stress (*mug187*) or during meiosis (*pab2* and *red1*), encoding proteins with similar localizations (*SPAC17H9.04c* and *rpm1*), or coding for proteins with related functions (*scw1*, *SPAC17H9.04c* and *rpm1*). This is consistent with the concept of the posttranscriptional operon or regulon, which states that RBPs coordinate the expression of genes with related functions at the posttranscriptional level [69], [70].

Perhaps the most surprising result from this work is the fact that only a relatively small fraction of genes (16.2%) is affected in the whole RBP deletion collection, suggesting that the function of non-essential RBPs is not sufficient to explain the large range of mRNA half-lives in fission yeast. There are several non-mutually exclusive explanations can account for this phenomenon. First, genetic redundancy may provide backup pathways that compensate for the lack of single RBPs. Second, it is possible that the majority of half-lives are determined by essential RBPs, which were not analysed in this study. Third, the general decay machinery might be able to interact with specifically with mRNAs in a gene-specific way independently of RBPs. Fourth, recent studies have revealed the existence of widespread connections between transcription and RNA degradation (reviewed in [31]), in which the effects of mutations affecting RNA decay is compensated by changes in transcription. However, we did not find any indication of this phenomenon in the three strains that we examined. Finally, promoter sequences can regulate RNA stability without involvement of *cis* elements on the mRNAs, presumably through the cotranslational recruitment of proteins to general mRNA features (such as the cap or the poly(A) tail) [71], [72]. Our work provides a framework that will allow the examination of these hypotheses in fission yeast and other organisms.

## Materials and Methods

### Fission yeast methods

Standard methods and media were used [73]. For transcriptome analysis cells were grown in Edinburgh Minimal Medium (EMM) at 32°C to a cell density of 8×10^6^ cells/ml. For spot assays cells were grown in yeast extract medium (YE) to a concentration of 8×10^6^ cells/ml, and plated in 10-fold dilutions. The following concentrations were used in YE agar plates: H_2_O_2_ at 1, 1.5 and 2 mM; CdSO_4_ at 0.3, 0.4, 0.5 and 0.6 mM; hydroxyurea at 5 and 10 mM; methyl benzimidazol-2-yl-carbamate (MBC) at 5 µg/ml; cycloheximide at 10, 20 and 30 µg/ml; methyl methanesulfonate (MMS) at 0.0027%; calcofluor at 0.5 and 1 mg/ml; caffeine at 5, 10 and 15 mM; KCl at 0.8 and 1 M. For growth in non-fermentable sources, glucose in YE was replaced with 2% galactose and 0.1% glucose. For assessment of mating and sporulation cells were grown on malt extract agar (MEA). All plates were incubated at 32°C. For testing temperature-sensitive growth, cells were plated on YE agar and incubated at either 20°C or 36°C. Table S11 lists all the strains used in this work. Cells were made homothallic (h^90^) and auxotrophic markers were removed by crossing before the experiments.

### Verification of deletion strains

The presence of a correct deletion was first assessed by examining the microarray signal for probes corresponding to the deleted gene. When this approach produced ambiguous results (for example, if a gene was expressed at low levels), we performed gene-specific diagnostic PCR. Overall, we verified the deletion for 74 strains and confirmed that the expected gene was not deleted in 12 others (Table S1).

### RNA preparation and microarray experiments

Total RNA was purified using phenol extraction [74]. Fluorescently labelled cDNA was prepared from total RNA using the SuperScript Plus Direct cDNA Labelling System (Life Technologies) as described by the manufacturer, except for the following modifications: 8 µg of total RNA was labelled in a reaction volume of 15 µl. 0.5 µl of 10× nucleotide mix with labelled nucleotide were used (1/3 of the recommended amount) and 1 µl of a home-made dNTP mix (0.5 mM dATP, 0.5 mM dCTP, 0.5 mM dGTP, 0.3 mM dTTP) was added to the reaction. All other components were used at the recommended concentrations. Note that these changes are essential to prevent dye-specific biases. Labelled cDNAs were hybridised to oligonucleotide microarrays manufactured by Agilent as described [63]. Microarrays were scanned with a GenePix 4000A microarray scanner and analysed with GenePix Pro 5.0 (Molecular Devices).

### Determination of mRNA stabilities

mRNA decay rates were determined using *in vivo* labelling with 4-thiouridine (4sU) [12]. Briefly, cells were grown in EMM at 32°C, and mRNAs were labelled by the addition of 4sU to the medium at a final concentration of 75 µg/ml. Cells were collected after incubation with 4sU for 7 or 10 minutes depending on the strain. An isogenic wild type was processed in parallel with each of the mutants. Total RNA was phenol-extracted and 4sU-labelled RNA was biotinylated and purified as described [12]. Finally, 4sU-labelled fractions and total RNA were compared using DNA microarrays as described above.

### RIP-chip experiments

Immunoprecipitation of TAP-tagged proteins was carried out using monoclonal antibodies against protein A (Sigma), and myc-tagged proteins were purified using the 9E11 monoclonal antibody (Abcam). For Scw1-TAP and Zfs1-TAP RIp-chips were performed as described [62] except for the following modifications: 1) the lysis buffer contained 1 mM PMSF and 1∶100 protease inhibitor cocktail (sigma P8340) and 2) magnetic beads containing the immunoprecipitate were resuspended in 50 µl of wash buffer containing 1 mM DTT, 1 unit/ml of SuperaseIN (Ambion) and 30 units/ml of AcTev protease (Life Technologies). The solution with the beads was incubated for 1 h at 19°C, the supernatant recovered and RNA extracted using PureLink RNA micro columns (Life Technologies) according to the manufacturer's instructions. The RNA was eluted from the column in 12 µl and used for labelling without amplification. For Red1-myc we followed a published protocol [62], except that the lysate was prepared in the following buffer: 10 mM Hepes pH 7.4, 100 mM KCl, 5 mM MgCl_2_, 25 mM EDTA, 0.5% NP-40, 1% Triton X-100, 0.1% SDS and 10% glycerol containing 1 mM PMSF and 1∶100 protease inhibitor cocktail (sigma P8340).

### Microarray data analysis

Microarray data for transcriptome analysis were normalized using Loess, and for RNA stability determination expression ratios were median-centred. Differentially expressed genes were identified using Significance Analysis of Microarrays (SAM) [37]. Significance of the overlap between gene sets was determined using Fisher's exact test.

Comparison with published microarray datasets was performed as follows: For *pab2* we used a list of 31 significantly up-regulated genes (Table 1 in reference [41]), for *red1* we used Tables S2 and S3, which report lists of 121 and 30 genes that are up-regulated or down-regulated, respectively, at least two-fold [42]. For *zfs1* we applied SAM to data from four microarrays from wild type cells and from *zfs1* mutants [39] using an FDR of 0. For the comparison with ncRNAs from *red1Δ* cells we used Table S2, which contains 269 ncRNAs [44].

### Analysis of RIp-chip experiments

The analysis of RIp-chip experiments was performed as described [12]. Briefly, all RNAs present in the immunoprecipitate were ranked by their enrichment levels (‘physical targets’). We then made lists of physical targets containing increasing amounts of genes (starting with the most enriched), and quantified the number of genes in each of the lists whose expression was affected in the corresponding RBP mutant. As a control, the analysis was repeated with a randomized list of physical targets. The initial gradient of the RIp-chip data is higher than that of the randomized data, indicating that the physical targets are enriched in genes affected by the mutation. When the gradient of both curves converges, the RIp-chip data stop having predictive value for the identification of regulated mRNAs. This point was chosen as a cut-off for the definition of RBP targets. RNAs identified in both biological repeats were selected. Significance of the overlap between gene sets was determined using Fisher's exact test.

### Identification of potential regulatory elements

We searched for enriched sequence elements in 5′-, 3′-UTRs and coding sequences using REFINE [60] with default parameters. The searches used sequence databases of UTRs and coding regions generated using information from GeneDB (http://old.genedb.org/), now PomBase (http://www.pombase.org/), on May 9, 2011 [36]. A threshold E value of 10^−8^ was used for selection of the motifs displayed in Table S8. Sequence logos were generated with WebLogo (http://weblogo.berkeley.edu/) [75].

### RNA quantification by qPCR

Wild type *leu1-32* or *red1Δ*::kanMX6 *leu1-32* cells were transformed with plasmids pRGT1-GFP-TTAAAC8x or pRGT1-GTAAAC8x [61]. Both plasmids express GFP under the control of the *adh1* promoter and contain 8 copies of the indicated sequence motifs. Cells were grown in EMM to a concentration of 10^7^ cells/ml. Total RNA was extracted using a hot phenol protocol [74]. 20 µg of total RNA were treated with 2 units of Turbo DNAse (Life Technologies) and purified using a PureLink RNA Micro kit (Life Technologies) following the manufacturer's protocol. 1 µg of purified RNA per sample was reverse-transcribed using Superscript III (Life Technologies). Quantitative analysis of RNA levels was performed using Sybr Green JumpStart Taq ReadyMix (Sigma) in a real-time PCR machine (Rotor-Q Gene, Quiagen) using the following program: 10 minutes at 95°C, then 40 cycles of 95°C for 10 seconds, 60°C for 15 seconds and 72°C for 30 seconds, with a final 5-second melting ramp of 1°C steps (from 50°C to 95°C) for acquisition. The following primers were used: GFP-F (CATCATGGCAGACAAACAAAA), GFP_R (AAAGGGCAGATTGTGTGGAC), ACT2_F (CCGGACTCGAGAAGAAACAT) and ACT2_R (AACCACCTTTTTCCGCTCTT). Quantification of relative levels was performed as follows: Ratio (GFP/actin) = (Eff∶GFP^-Ct∶GFP^)/(Eff∶actin^-Ct∶actin^), where Eff∶GFP and Eff∶actin represent the amplification efficiencies, and Ct∶GFP and Ct∶actin are the critical cycles.

### Accession numbers

A total of 219 microarray experiments were performed. All microarray and sequencing data have been deposited in ArrayExpress with accession numbers E-MTAB-2314 (microarray expression experiments), E-MTAB-2317, E-MTAB-2318 and E-MTAB-2712 (stability data), E-MTAB-2709 (RIp-chip experiments) and RNA-seq of splicing mutants (E-MTAB-2695).

## Supporting Information

Figure S1 Comparisons with published data. (A) Overlap between mRNAs up-regulated in zfs1 mutants reported in this work and published data. The number in brackets shows the expected overlap if randomly-generated lists of the corresponding sizes were used. The p value of the observed overlap is shown on the right side. (B) As in A, comparison for up-regulated mRNAs from cells with mutations in pab2. (C) As in A, comparing up-regulated mRNAs from red1Δ cells. (D) As in A, for ncRNAs up-regulated in red1Δ cells.
(PDF)

Figure S2 RNA-seq analysis of splicing mutants. (A) Two models to explain an accumulation of intronic reads in an RBP mutant. Intronic reads are shown in green and reads that span exon-intron junctions (EIJs) in blue. (B) Ratio of intronic and EIJ reads between SPBC18H10.07Δ and wild type cells. The data are shown for the 100 introns that displayed the highest accumulation of intronic reads in the mutant. Note the concomitant accumulation of both types of read, indicating that pre-mRNAs accumulate. (C) Overlap between introns accumulated in SPBC18H10.07Δ mutants detected by RNA-seq and microarray experiments. The number in brackets corresponds to the expected overlap if randomly-generated lists of the corresponding sizes were used. The p value of the observed overlap is shown under the Venn diagram. (D) As in B, for SPAC30D11.14c. (E) As in C, for SPAC30D11.14c.
(PDF)

Figure S3 Correlations between changes in mRNA stability and mRNA levels. (A) Overlap between mRNAs up-regulated and stabilized in red1 mutants. The number in brackets corresponds to the expected overlap if randomly-generated lists of the corresponding sizes were used. The p value of the observed overlap is shown on the right side. (B) As in A, comparing mRNAs up-regulated and stabilized in pab2Δ cells. (C) As in A, comparing mRNAs up-regulated and stabilized in rnc1 cells. (D) As in A, for mRNAs up-regulated and stabilized in csx1 cells.
(PDF)

Figure S4 RIp-chip experiments. (A) Selection of functionally relevant Red1-associated transcripts (see Materials and Methods for details). Red line: the number of genes selected from a ranked list of RIp-chip enrichments (x axis) is plotted against the number of genes in the list whose expression levels are increased in red1Δ cells (y axis). Dashed line: rate at which genes overexpressed in red1Δ cells would be expected to be found if chosen randomly from the list of enriched genes. The point at which the slope of the red line decreases and becomes closer to the random curve (arrow) was used to define functionally relevant Red1 targets. (B) As A, for Zfs1. (C) Overlap between RNAs bound to Red1 and RNAs up-regulated (left) or down-regulated (right) in red1 mutants. The number in brackets corresponds to the expected overlap if randomly-generated lists of the corresponding sizes were used. The p value of the observed overlap is shown under the Venn diagram. (D) As C, for Zfs1. (E) As D, for Scw1.
(PDF)

Figure S5 Phenotypic characterization of RBP deletion mutants. (A) Number of phenotypes per strain. The bar chart shows the number of strains that displayed phenotypes in the indicated number of conditions. (B) Examples of phenotype assays. Wild type and mutant cells were plated in the indicated conditions (see methods for details).
(PDF)

Figure S6 Examples of sporulation defects. Wild type cells or the indicated mutants were incubated on malt extract plates to induce sexual differentiation. Sporulation defects have been reported for meu5 (Amorim et al. 2010 Mol Sys Biol 6:380) and mug28 (Shigehisa et al. 2010 Mol Biol Cell 21:1955).
(PDF)

Table S1 RNA-binding proteins in fission yeast.
(XLSX)

Table S2 Up-regulated mRNAs.
(XLSX)

Table S3 Up-regulated mRNAs.
(XLSX)

Table S4 Gene enrichments.
(XLSX)

Table S5 Up-regulated ncRNAs.
(XLSX)

Table S6 Up-regulated introns.
(XLSX)

Table S7 RNA half-lives.
(XLSX)

Table S8 Potential regulatory motifs.
(XLSX)

Table S9 RNAs identified in RIp-chip experiments.
(XLSX)

Table S10 Phenotypic characterization of RBP deletion mutants.
(XLSX)

Table S11 Strains used in this work.
(XLSX)
