# Supplementary material for: Systematic Analysis of the Role of RNA-Binding Proteins in the Regulation of RNA Stability
Source: PLoS Genet. 2014 Nov 6;10(11):e1004684. doi: 10.1371/journal.pgen.1004684 (PMC4222612; doi:10.1371/journal.pgen.1004684)
Supplement: Figure S1 — Comparisons with published data. (A) Overlap between mRNAs up-regulated in zfs1 mutants reported in this work and published data. The number in brackets shows the expected overlap if randomly-generated lists of the corresponding sizes were used. The p value of the observed overlap is shown on the right side. (B) As in A, comparison for up-regulated mRNAs from cells with mutations in pab2. (C) As in A, comparing up-regulated mRNAs from red1Δ cells. (D) As in A, for ncRNAs up-regulated in red1Δ cells. (PDF) [file pgen.1004684.s001.pdf]

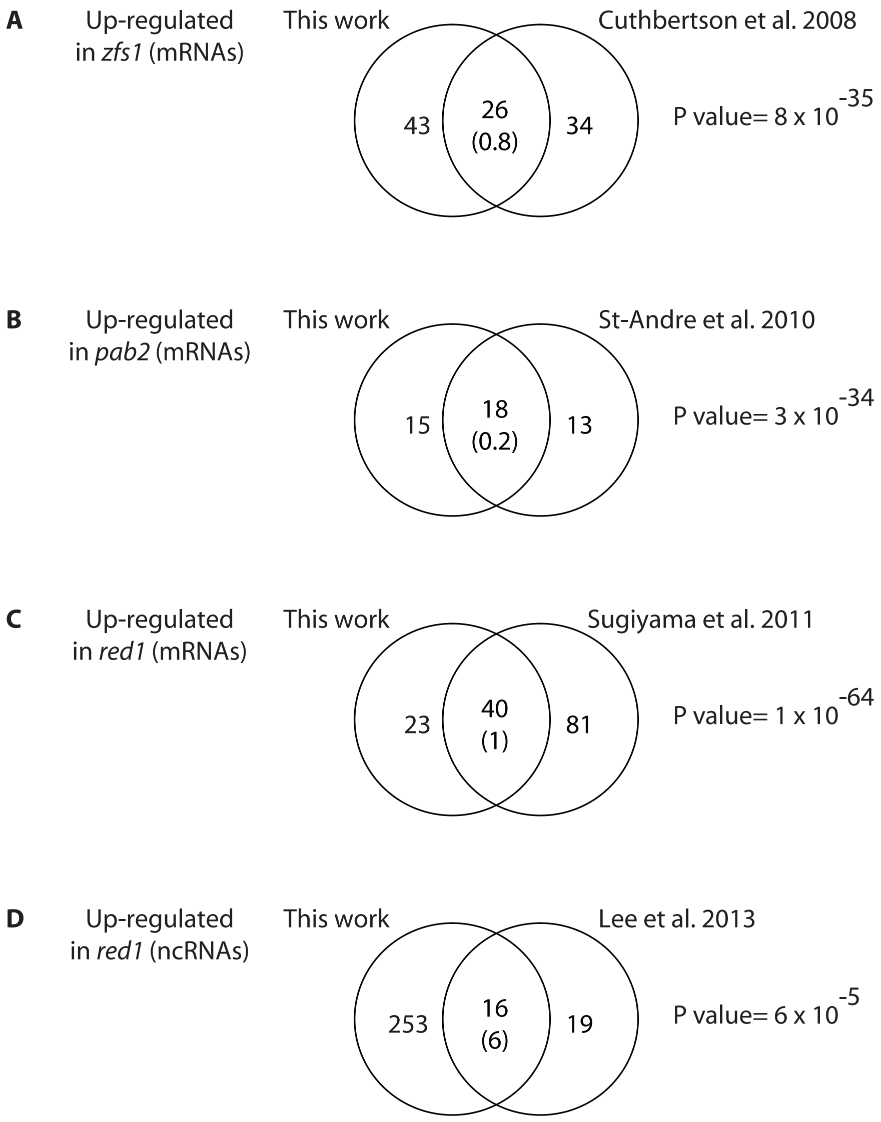

Figure S1. Comparisons with published data.

(A) Overlap between mRNAs up-regulated in *zfs1* mutants reported in this work and published data. The number in brackets shows the expected overlap if randomly-generated lists of the corresponding sizes were used. The p value of the observed overlap is shown on the right side. (B) As in A, comparison for up-regulated mRNAs from cells with mutations in *pab2*. (C) As in A, comparing up-regulated mRNAs from *red1Δ* cells. (D) As in A, for ncRNAs up-regulated in *red1Δ* cells.
