# Supplementary material for: Systematic Analysis of the Role of RNA-Binding Proteins in the Regulation of RNA Stability
Source: PLoS Genet. 2014 Nov 6;10(11):e1004684. doi: 10.1371/journal.pgen.1004684 (PMC4222612; doi:10.1371/journal.pgen.1004684)
Supplement: Figure S2 — RNA-seq analysis of splicing mutants. (A) Two models to explain an accumulation of intronic reads in an RBP mutant. Intronic reads are shown in green and reads that span exon-intron junctions (EIJs) in blue. (B) Ratio of intronic and EIJ reads between SPBC18H10.07Δ and wild type cells. The data are shown for the 100 introns that displayed the highest accumulation of intronic reads in the mutant. Note the concomitant accumulation of both types of read, indicating that pre-mRNAs accumulate. (C) Overlap between introns accumulated in SPBC18H10.07Δ mutants detected by RNA-seq and microarray experiments. The number in brackets corresponds to the expected overlap if randomly-generated lists of the corresponding sizes were used. The p value of the observed overlap is shown under the Venn diagram. (D) As in B, for SPAC30D11.14c. (E) As in C, for SPAC30D11.14c. (PDF) [file pgen.1004684.s002.pdf]

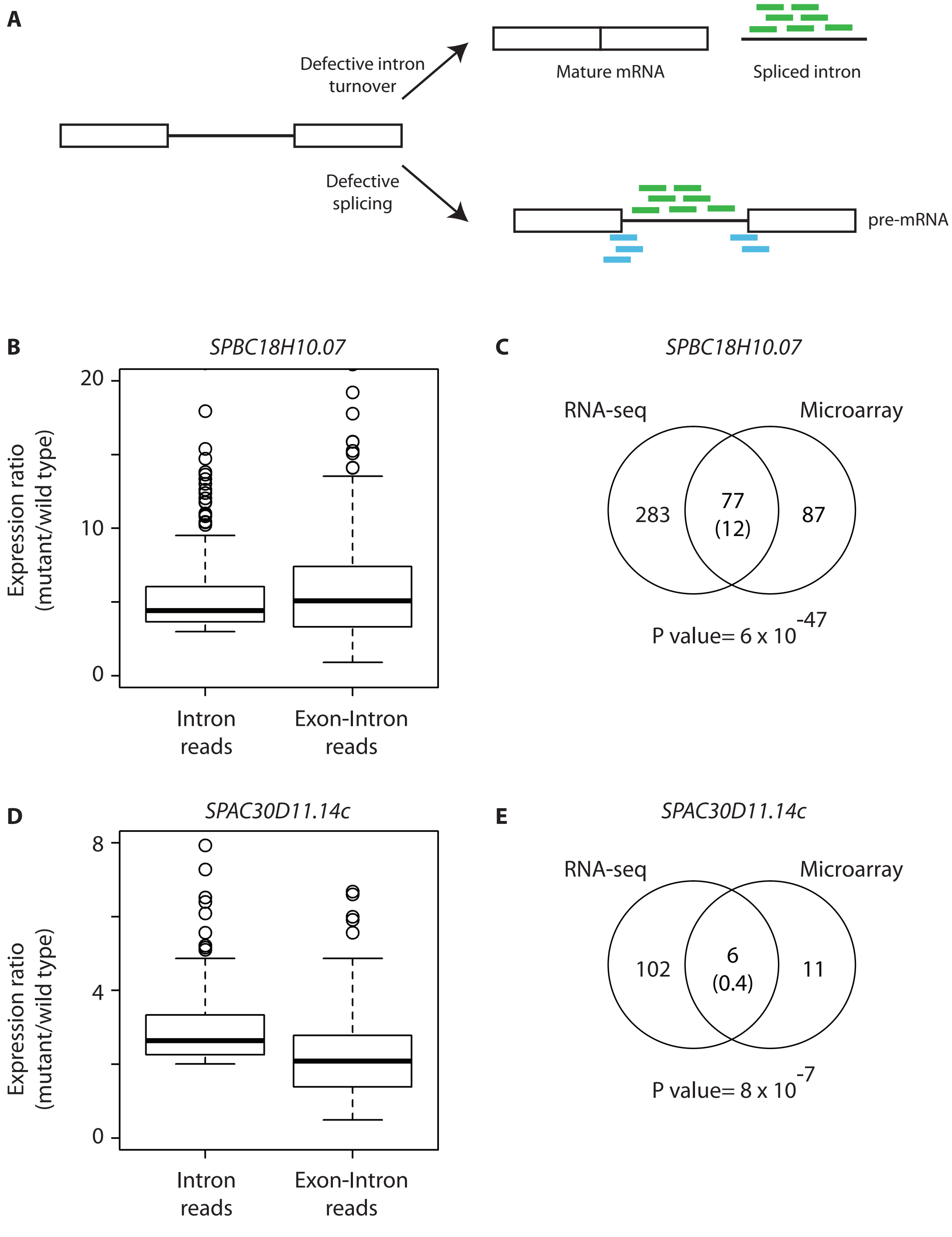

Figure S2. RNA-seq analysis of splicing mutants.

(A) Two models to explain an accumulation of intronic reads in an RBP mutant. Intronic reads are shown in green and reads that span exon-intron junctions (EIJs) in blue. (B) Ratio of intronic and EIJ reads between *SPBC18H10.07Δ* and wild type cells. The data are shown for the 100 introns that displayed the highest accumulation of intronic reads in the mutant. Note the concomitant accumulation of both types of read, indicating that pre-mRNAs accumulate. (C) Overlap between introns accumulated in *SPBC18H10.07Δ* mutants detected by RNA-seq and microarray experiments. The number in brackets corresponds to the expected overlap if randomly-generated lists of the corresponding sizes were used. The p value of the observed overlap is shown under the Venn diagram. (D) As in B, for *SPAC30D11.14c*. (E) As in C, for *SPAC30D11.14c.*
