# Supplementary material for: Systematic Analysis of the Role of RNA-Binding Proteins in the Regulation of RNA Stability
Source: PLoS Genet. 2014 Nov 6;10(11):e1004684. doi: 10.1371/journal.pgen.1004684 (PMC4222612; doi:10.1371/journal.pgen.1004684)
Supplement: Figure S3 — Correlations between changes in mRNA stability and mRNA levels. (A) Overlap between mRNAs up-regulated and stabilized in red1 mutants. The number in brackets corresponds to the expected overlap if randomly-generated lists of the corresponding sizes were used. The p value of the observed overlap is shown on the right side. (B) As in A, comparing mRNAs up-regulated and stabilized in pab2Δ cells. (C) As in A, comparing mRNAs up-regulated and stabilized in rnc1 cells. (D) As in A, for mRNAs up-regulated and stabilized in csx1 cells. (PDF) [file pgen.1004684.s003.pdf]

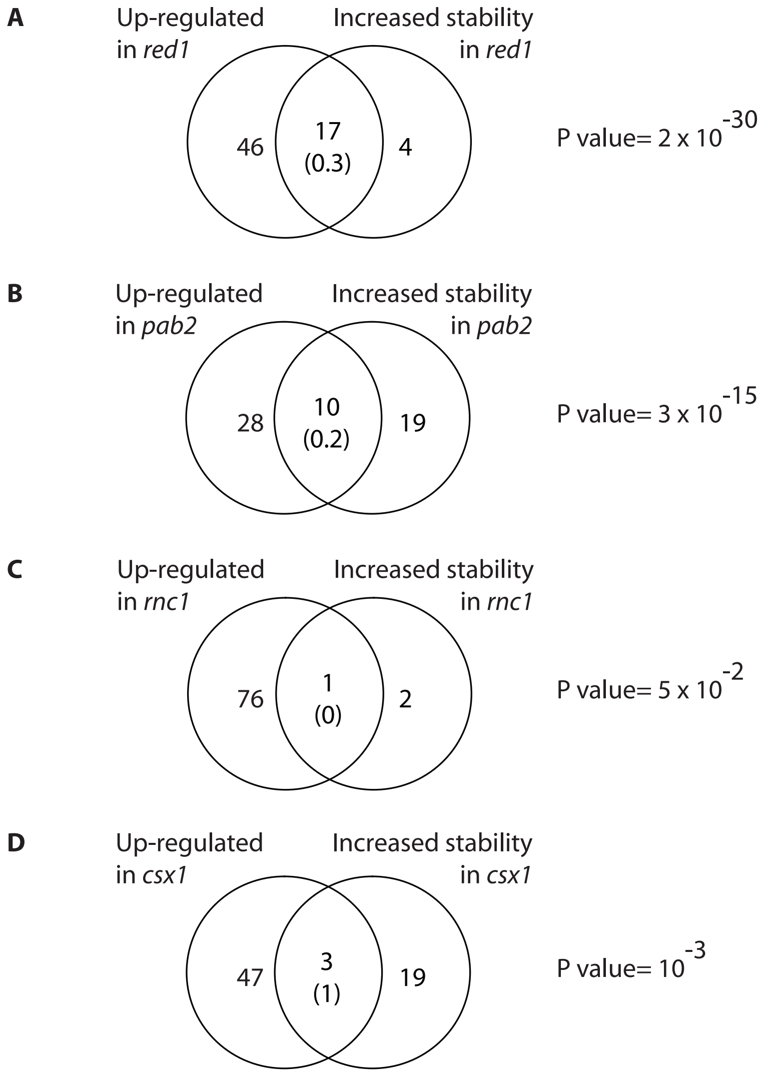

Figure S3. Correlations between changes in mRNA stability and mRNA levels.

(A) Overlap between mRNAs up-regulated and stabilized in *red1* mutants. The number in brackets corresponds to the expected overlap if randomly-generated lists of the corresponding sizes were used. The p value of the observed overlap is shown on the right side. (B) As in A, comparing mRNAs up-regulated and stabilized in *pab2Δ* cells. (C) As in A, comparing mRNAs up-regulated and stabilized in *rnc1* cells. (D) As in A, for mRNAs up-regulated and stabilized in *csx1* cells.
