# Supplementary material for: Systematic Analysis of the Role of RNA-Binding Proteins in the Regulation of RNA Stability
Source: PLoS Genet. 2014 Nov 6;10(11):e1004684. doi: 10.1371/journal.pgen.1004684 (PMC4222612; doi:10.1371/journal.pgen.1004684)
Supplement: Figure S4 — RIp-chip experiments. (A) Selection of functionally relevant Red1-associated transcripts (see Materials and Methods for details). Red line: the number of genes selected from a ranked list of RIp-chip enrichments (x axis) is plotted against the number of genes in the list whose expression levels are increased in red1Δ cells (y axis). Dashed line: rate at which genes overexpressed in red1Δ cells would be expected to be found if chosen randomly from the list of enriched genes. The point at which the slope of the red line decreases and becomes closer to the random curve (arrow) was used to define functionally relevant Red1 targets. (B) As A, for Zfs1. (C) Overlap between RNAs bound to Red1 and RNAs up-regulated (left) or down-regulated (right) in red1 mutants. The number in brackets corresponds to the expected overlap if randomly-generated lists of the corresponding sizes were used. The p value of the observed overlap is shown under the Venn diagram. (D) As C, for Zfs1. (E) As D, for Scw1. (PDF) [file pgen.1004684.s004.pdf]

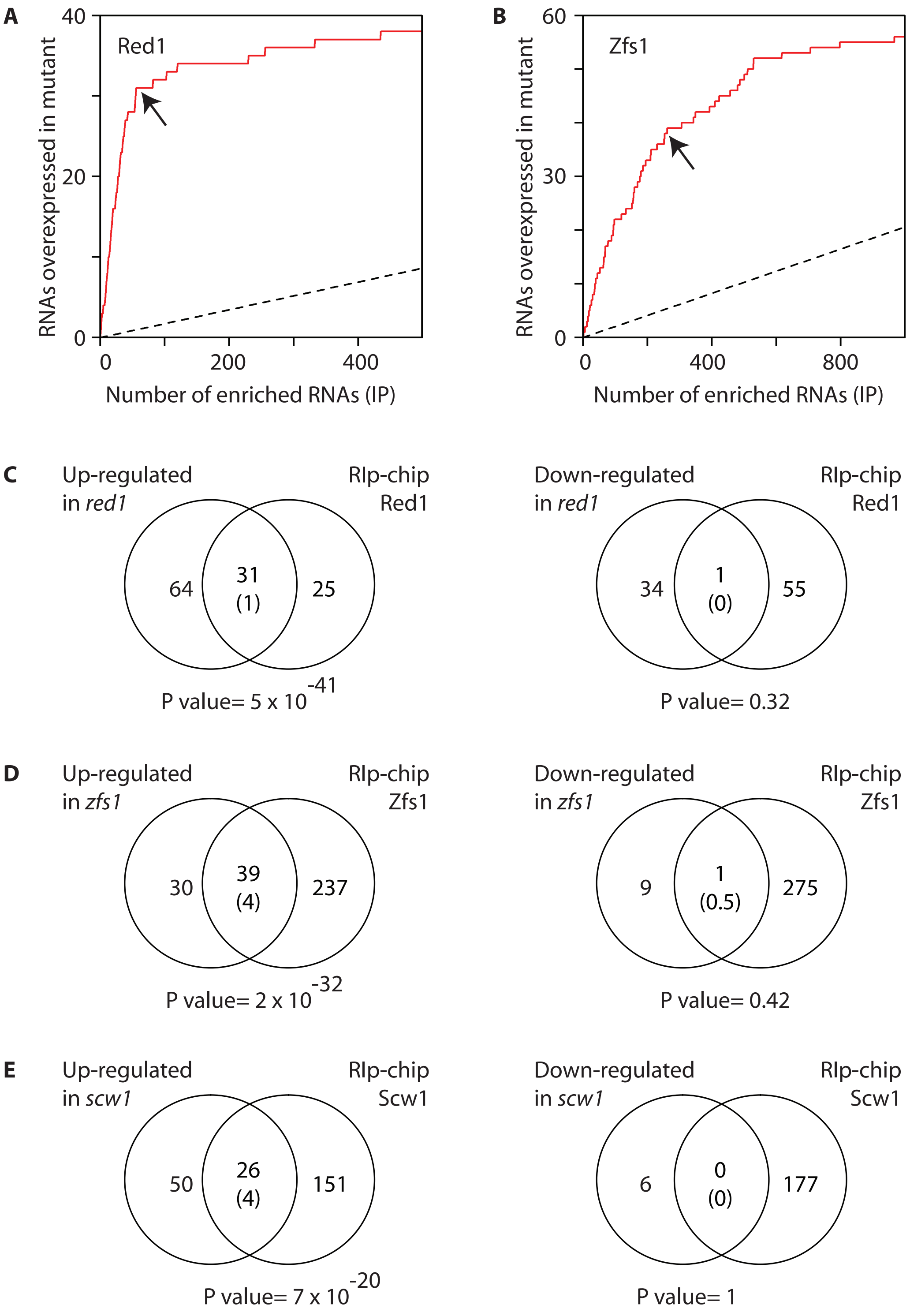

Figure S4. RIp-chip experiments.

(A) Selection of functionally relevant Red1-associated transcripts (see Materials and Methods for details). Red line: the number of genes selected from a ranked list of RIp-chip enrichments (x axis) is plotted against the number of genes in the list whose expression levels are increased in *red1Δ* cells (y axis). Dashed line: rate at which genes overexpressed in *red1Δ* cells would be expected to be found if chosen randomly from the list of enriched genes. The point at which the slope of the red line decreases and becomes closer to the random curve (arrow) was used to define functionally relevant Red1 targets. (B) As A, for Zfs1. (C) Overlap between RNAs bound to Red1 and RNAs up-regulated (left) or down-regulated (right) in *red1* mutants. The number in brackets corresponds to the expected overlap if randomly-generated lists of the corresponding sizes were used. The p value of the observed overlap is shown under the Venn diagram. (D) As C, for Zfs1. (E) As D, for Scw1.
