# Supplementary material for: Systematic Analysis of the Role of RNA-Binding Proteins in the Regulation of RNA Stability
Source: PLoS Genet. 2014 Nov 6;10(11):e1004684. doi: 10.1371/journal.pgen.1004684 (PMC4222612; doi:10.1371/journal.pgen.1004684)
Supplement: Figure S5 — Phenotypic characterization of RBP deletion mutants. (A) Number of phenotypes per strain. The bar chart shows the number of strains that displayed phenotypes in the indicated number of conditions. (B) Examples of phenotype assays. Wild type and mutant cells were plated in the indicated conditions (see methods for details). (PDF) [file pgen.1004684.s005.pdf]

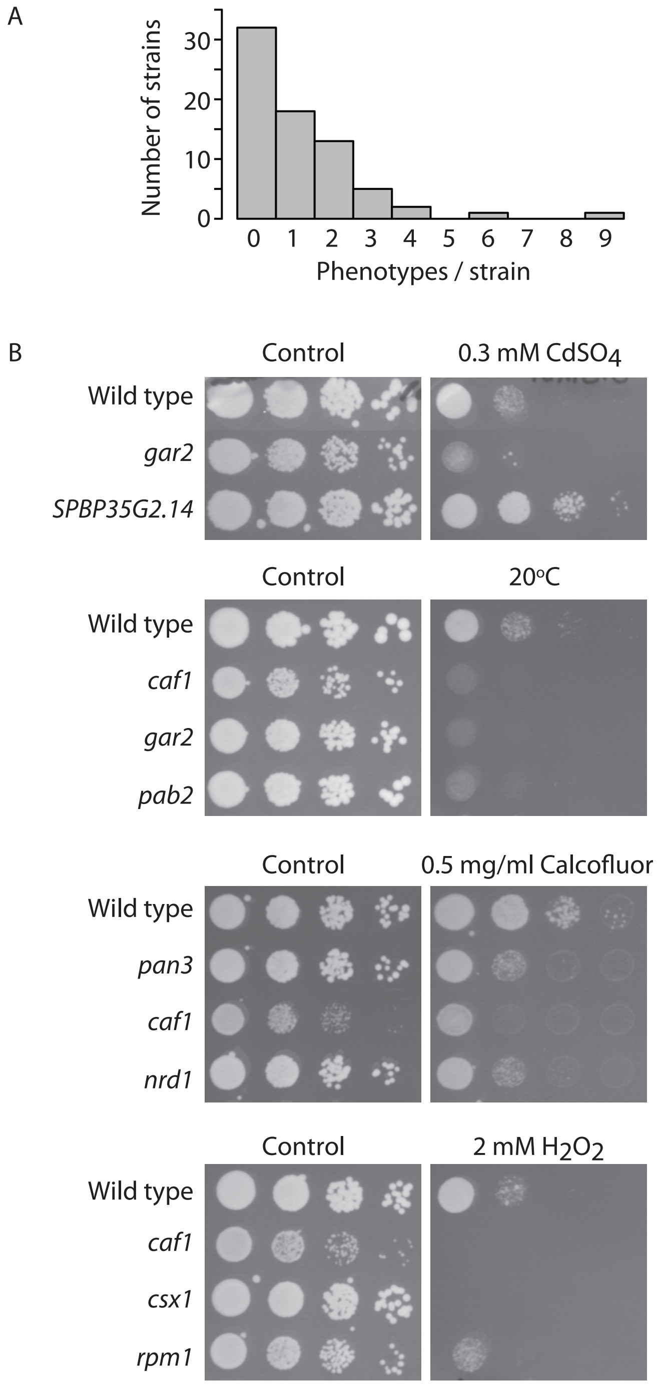

Figure S5. Phenotypic characterization of RBP deletion mutants.

(A) Number of phenotypes per strain. The bar chart shows the number of strains that displayed phenotypes in the indicated number of conditions. (B) Examples of phenotype assays. Wild type and mutant cells were plated in the indicated conditions (see methods for details).
