# Supplementary material for: Systematic Analysis of the Role of RNA-Binding Proteins in the Regulation of RNA Stability
Source: PLoS Genet. 2014 Nov 6;10(11):e1004684. doi: 10.1371/journal.pgen.1004684 (PMC4222612; doi:10.1371/journal.pgen.1004684)
Supplement: Figure S6 — Examples of sporulation defects. Wild type cells or the indicated mutants were incubated on malt extract plates to induce sexual differentiation. Sporulation defects have been reported for meu5 (Amorim et al. 2010 Mol Sys Biol 6:380) and mug28 (Shigehisa et al. 2010 Mol Biol Cell 21:1955). (PDF) [file pgen.1004684.s006.pdf]

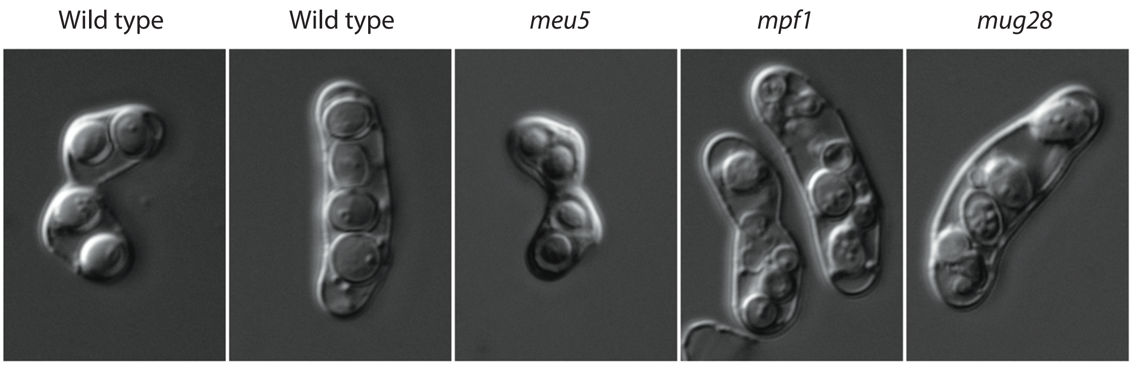

Figure S6. Examples of sporulation defects.

Wild type cells or the indicated mutants were incubated on malt extract plates to induce sexual differentiation. Sporulation defects have been reported for *meu5* (Amorim *et al*. 2010 Mol Sys Biol 6:380) and *mug28* (Shigehisa *et al*. 2010 Mol Biol Cell 21:1955).
